# Coupling of radiofrequency with magnetic nanoparticles treatment as an alternative physical antibacterial strategy against multiple drug resistant bacteria

**DOI:** 10.1038/srep33662

**Published:** 2016-09-27

**Authors:** Akhilesh K. Chaurasia, Nanasaheb D. Thorat, Anshula Tandon, Jin-Hahn Kim, Sung Ha Park, Kyeong Kyu Kim

**Affiliations:** 1 Department of Molecular Cell Biology, Sungkyunkwan University School of Medicine, Suwon 16419, Korea; 2 Departments of Physics, Sungkyunkwan University, Suwon 16419, Korea; 3 Sungkyunkwan Advanced Institute of Nanotechnology (SAINT), Sungkyunkwan University, Suwon 16419, Korea

## Abstract

Antibiotic resistant bacteria not only affect human health and but also threatens the safety in hospitals and among communities. However, the emergence of drug resistant bacteria is inevitable due to evolutionary selection as a consequence of indiscriminate antibiotic usage. Therefore, it is necessary to develop a novel strategy by which pathogenic bacteria can be eliminated without triggering resistance. We propose a novel magnetic nanoparticle-based physical treatment against pathogenic bacteria, which blocks biofilm formation and kills bacteria. In this approach, multiple drug resistant *Staphylococcus aureus* USA300 and uropathogenic *Escherichia coli* CFT073 are trapped to the positively charged magnetic core-shell nanoparticles (MCSNPs) by electrostatic interaction. All the trapped bacteria can be completely killed within 30 min owing to the loss of membrane potential and dysfunction of membrane-associated complexes when exposed to the radiofrequency current. These results indicate that MCSNP-based physical treatment can be an alternative antibacterial strategy without leading to antibiotic resistance, and can be used for many purposes including environmental and therapeutic applications.

In pathogenic bacteria, antibiotic resistance is ever increasing against most of the currently used antibiotic drugs, thus, posing a serious threat to human health^1^. Growing concerns of multiple drug resistance (MDR) alarmed the international scientific community to deal with MDR threat^2^,^3^,^4^. The first World Health Organization (WHO) global report on antibiotic resistance published in 2014 specified that, “This serious threat is no longer a prediction for the future, it is happening right now in every region of the world and has the potential to affect anyone, of any age, in any country.” The development of antibiotic resistance is an inevitable phenomenon since it is the result of a gradual and continuous evolutionary and natural selection process. However, the excessive use and abuse of antibiotics in hospitals, animal rearing, and communities expedites the development of multiple drug resistant bacteria (MDRB). The MDR problem is further intensified by horizontal transfer of antibiotic resistance genes among heterogeneous microbial populations^5^. Accordingly, a rapid increase in MDRB population not only threatens human health but also causes an environmental crisis. For example, the contamination of aquatic ecosystems and water reservoirs with antibiotics causes the occurrence of MDRB at an alarming level and directly results in increasing infection rates^6^,^7^.

Antibiotic-based chemical therapy not only generates MDR by intrinsic mechanisms^8^, but also induces the biofilm formation as a survival strategy against antibiotic stress^9^. Biofilm formation against antibiotics is considered as one of the most dangerous microbial phenotypes and is the primary cause of chronic infections, which at times cannot be treated with an entire class of antibiotics^10^,^11^. For these reasons, a WHO study reports that there may be another 1–2 decades left for people to use the existing antibiotics and thereafter; the infections caused by MDRB will not be curable using these antibiotics. Therefore, there is an urgent requirement to develop a novel antibacterial strategy that does not trigger resistance but is still effective in eliminating pathogenic bacteria and their biofilm.

As an alternative to overcome the problems associated with antibiotic usage and the resultant drug resistance, nanoparticle (NP) based antibacterial therapies have received increasing attention^12^,^13^. The most intensively studied antibacterial nanomaterial is the metallic silver nanoparticle that demonstrates antimicrobial effect against various bacterial pathogens^14^. However, environmental concerns of silver nanoparticle (Ag-NP) persistence and toxicity limited its usage^15^. The toxicity of various metallic NPs is caused by the uncontrolled generation of reactive oxygen species through Fenton, Fenton-like, and Haber-Weiss reactions^16^. In addition, prolonged exposure to some NPs also produces adverse toxic effects^17^,^18^. To avoid the problems caused by long-term exposure, photothermal treatments have been developed. In this approach, gold nanoparticle (Au-NPs) were treated with near-infrared (NIR) light for heat-inactivation of bacteria^19^. However, due to low tissue penetration, NIR applications are limited. Therefore, development of an efficient NP-based antibacterial treatment is necessary to overcome current limitations of NP-based approaches.

In this study, we propose the exposure of MDRB trapped by magnetic core shell nanoparticles (MCSNPs) to radiofrequency (RF) current as an alternative to the usage of chemical antibiotics against bacteria. MCSNPs used in this study consisted of an Fe_3_O_4_ core and SiO_2_–NH_2_ shell (Fe_3_O_4_@SiO_2_-NH_2_). Fe_3_O_4_ is the least toxic magnetic nanoparticle (MNP) among other studied metal oxide NPs due to its intrinsic peroxidase-like activities^20^, and thus it is clinically used as a contrast agent in magnetic resonance imaging (MRI)^21^. We demonstrated that bacteria can be effectively trapped using MCSNP, and that their biofilm formation can be efficiently inhibited by MCSNPs. Furthermore, we showed that MCSNP-trapped bacteria can be entirely eliminated within 30 min when treated with MCSNPs under RF current using capacitive electric transfer (CET) devices. RF has previously been used in the treatment of many diseases such as cancer, depression, and chronic pain since it is not harmful and can deeply penetrate human tissues^22^,^23^,^24^,^25^. Particularly, medium frequency RF (0.3–3.0 MHz) generated from CET instrument is widely used for cosmetic applications^26^, alongside hyperthermia with metallic nanoparticles^24^. We also elucidated that bacteria is killed due to the loss of membrane potential followed by dysfunction of membrane-associated complexes responsible for bacterial bioenergetics since MCSNP bound to bacterial cell surface adversely affects the membrane when it stimulated by RF. These results suggest the possibility of applying RF coupled with MCSNP-based treatment (RMT) as a physical antibacterial strategy for medical and environmental purposes.

## Results and Discussion

For the purpose of trapping bacteria and subsequent capturing of bacteria-MCSNPs complex using external magnetic field, we developed a paramagnetic iron oxide core coated with an aminated silica shell. For the following reasons, the proposed MCSNP has several advantages over its bare counterpart in terms of antibacterial materials: (**a**) prevents agglomeration of the iron core NPs; (**b**) acts as physical barrier at the nano-bio interface to prevent the biological reduction of Fe(III) to Fe(II) using NADPH, thereby reducing ROS generation by Fenton, Fenton-like, and/or Haber-Weiss reactions; (**c**) prevents the passive internalization of NPs to bacteria by increasing the size (>40 nm); and (**d**) provides a homogeneous interaction surface for the negatively charged microbial cell.

**Figure 1:**
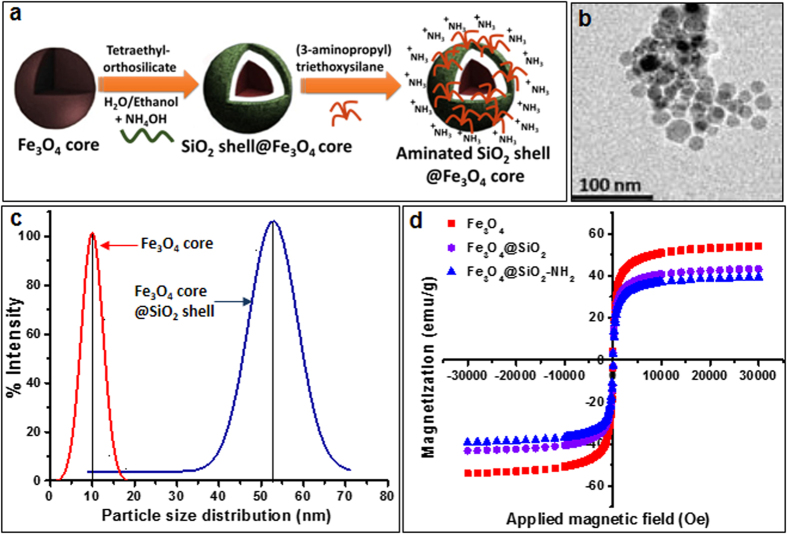
Synthesis of Fe_3_O_4_@SiO_2_-NH_2_ magnetic core-shell nanoparticles (MCSNPs) and characterization of their physicochemical properties. (**a**) Schematic diagram showing the steps for synthesizing the Fe_3_O_4_ magnetic core, surface coating with SiO_2_ shell, and functionalization with amine group (-NH_2_). (**b**) TEM image of a bare Fe_3_O_4_ nanoparticle core showing a diameter close to 10 nm. (**c**) Evaluation of particle size and distribution by dynamic light scattering. The average size of the bare Fe_3_O_4_ magnetic core is 10 nm in diameter while the average size of the final amine functionalized Fe_3_O_4_@SiO_2_ particle is increased up to 53 nm. (**d**) Magnetization properties of the Fe_3_O_4_ core alone, Fe_3_O_4_ core with silica coating (Fe_3_O_4_@SiO_2_), and amine functionalized Fe_3_O_4_@SiO_2_ -NH_2_ (MCSNP) particles were 55.0, 40.9, and 38.8 emu/g, respectively.

The iron oxide core (Fe_3_O_4_) coated with silica shell was synthesized, and subsequently the silicon shell was functionalized by conjugating amine groups as described as a schematic diagram in Fig. 1a, corresponding method and supporting information. The synthesis of the Fe_3_O_4_ core was verified by detection of peaks at 723 and 710 eV using X-ray photoelectron spectroscopy (XPS) analysis, which corresponds to the energy at 2p_3/2_ and 2p_1/2_ of pure Fe_3_O_4_ (Supplementary Figure S1A). Upon silica coating, the intensity of Fe electronic configuration peaks was decreased and the characteristic peak of Si_2p_ (Si-O) was seen at 103 eV (Supplementary Figure S1B). Finally, functionalized amine (-NH_2_) on the surface of Fe_3_O_4_–SiO_2_ core-shell was confirmed by a peak at 399 eV (Supplementary Figure S1B). Exact intensities of each peak corresponding to Si, O, Fe and amine groups were confirmed by X-Ray photoelectron spectroscopy (Supplementary Figure S1C-F). The TEM imaging showed that the size of Fe_3_O_4_ cores range between 10 to 15 nm (Fig. 1b). Dynamic light scattering (DLS) measurements showed that the average size distribution of Fe_3_O_4_ core NP was increased from 10 nm to 53 nm in diameter upon silica coating (Fig. 1c). The magnetization of bare Fe_3_O_4_ core was 55 emu/g and was decreased up to 40.90 emu/g and 37.81 emu/g after SiO_2_ and NH_2_ coating, respectively (Fig. 1d), suggesting the net magnetization of core Fe_3_O_4_ was affected by the coating of non-magnetic molecules. Zeta potential of NPs was examined at each synthesis stages, and Fe_3_O_4_@SiO_2_-NH_2_ in PBS buffer (pH 7.4) was confirmed to be 7.2 (Supplementary Figure S2A,B), suggesting that MCSNPs maintain a positive charge at physiological pH (pH 7.4), which is important for trapping bacteria with negative surface charge^27^.

**Figure 2:**
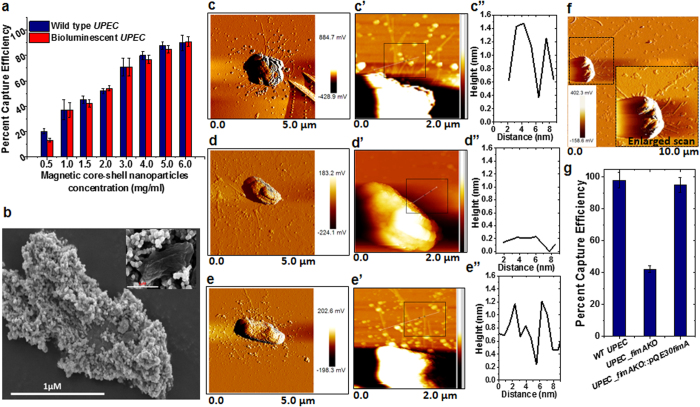
Magnetic trapping of bacteria using MCSNPs, and AFM images of *UPEC*. **(a)** Magnetic trapping efficiency of *UPEC* MDRB treated at different concentrations of MCSNPs was assessed by measuring the optical density (OD_600_) and bioluminescence of free bacteria after magnetic field separation. **(b)** SEM images of MCSNP-trapped *UPEC* when 6 mg/mL of MCSNP was incubated with 1 × 10^8^ bacteria. The inset shows the SEM image when bacteria were treated with 1 mg/mL MCSNP. **(c–e)** AFM amplitude images of *UPEC* for the assessment of phenotype of wild type *UPEC* (**c**), ∆*fimA* deletion-insertion mutant (**d**), and complementation strains (**e**). AFM images of *UPEC* strains show the presence of numerous fimbriae in the wild type *UPEC,* absence of fimbriae in the *∆fimA* deletion-insertion mutant (∆*fimAΩkm*^*r*^), and the restoration of fimbriae in the complemented strain of *∆fimA* knockout (∆*fimAΩkm*^*r*^*::pQE30fimA*). **(c’–e’)** AFM images of the same samples in (**c–e**) in height mode. The diameter of fimbriae in black box is displayed in figure (**c”–e”**). Note that *∆fimA* knockout strain shows no distinct fibril due to the absence of fimbriae (**d”**). (**f**) AFM images of 10 × 10 μm^2^ scan shows MCSNPs bound to fimbriae of *UPEC*, inset shows enlarged scan (5 × 5 μm^2^). (**g**) MCSNP-mediated trapping and capture efficiencies of WT, *∆fimA* knockout, and complemented *UPEC* strains show that the capturing efficiency of the knockout strain is 55% lower than the wild type and complemented strains.

The trapping efficiency of MCSNP for MDRB was evaluated using multidrug resistant uropathogenic *Escherichia coli* CFT073 bacteria (hereafter referred as *UPEC*). For visualization of MCSNP-mediated bacterial trapping, genes responsible for bioluminescence, *luxCDABE,* were inserted in the *UPEC* strains (Supplementary Figure S3, Tables S1 and S2). Wild type (WT) or genetically engineered bioluminescent bacteria were mixed with varying concentrations of MCSNPs for 10 minutes, and then the MCSNP-trapped bacteria were captured using an external magnetic field. The amount of remaining free bacteria in solution was quantified by measuring the OD_600_ or bioluminescence. The capture efficiency for *UPEC* reached 90.1% when 6 mg/mL MCSNPs were mixed with 10^8^ bacteria (60 pg per cell equivalent) in 1 mL PBS buffer (pH 7.4) (Fig. 2a). The trapping efficiency was further evaluated using bioluminescent *UPEC* bacteria and was found to be consistent with the optical density measurement (Fig. 2a). To further investigate binding of MCSNPs to the bacterial cell surface, the MCSNP treated cells were examined using scanning electron microscopy (SEM) (Fig. 2b). The SEM images of *UPEC* clearly showed the binding of MCSNPs on the bacterial surface, presumably due to electrostatic interaction of the positively charged MCSNPs with negatively charged *UPEC* cell surface (Fig. 2b). SEM image analysis of MCSNPs bound to bacteria showed that MCSNPs are closely packed on the outer membrane of *UPEC* (Fig. 2b and inset). The electrostatic attachment of numerous MCSNPs at the bacterial surface makes them responsive towards an external magnetic field, which enables the separation of the MCSNP-bound bacteria from free bacteria. In addition, it is also expected that most MCSNPs in the current study, with the size of 53 nm, remained on the surface instead of entering into the cells, because it is known that NPs bigger than 40 nm cannot penetrate the cell membrane passively^18^. These physicochemical properties of MCSNPs can be applied to reduce contaminating MDRB pathogens in water to negligible levels by MCSNP-mediated bacterial-trapping and facile magnetic capture, which is useful to avoid any contamination with bacterial toxins and other harmful bacterial components.

The binding mode of MCSNPs to Gram-negative bacteria was further investigated by examining the interaction of MCSNP with fimbriae, a major cell surface appendage in *UPEC. UPEC* possess a large number of type I fimbriae, which play an important role in biofilm formation and primary interaction with host cells during invasion^28^,^29^. Therefore, we hypothesized that fimbriae are one of major binding sites of MCSNPs on the outer membrane. To investigate the interaction between fimbriae and MCSNP, the *fimA* gene that encodes fimbriae was deleted by replacing it with a kanamycin cassette using the red recombinase system (Supplementary Figure 4A). The genotypes of the *fimA* knockout (∆*fimA*) and ∆*fimA* complemented strains (*∆fimA::pQE30fimA*) were characterized by confirming the absence of *fimA* gene in ∆*fimA* strain and presence of *fimA* gene in *∆fimA::pQE30fimA* strain using appropriate primer pairs and PCR reactions (Supplementary Figure 4B and Tables S1 and S2). To investigate the phenotypes of the wild type, ∆*fimA,* and *∆fimA::pQE30fimA UPEC* strains, their cell surface structures were assessed by atomic force microscopy (AFM) (Fig. 2c–e). In an amplitude mode, the AFM images of the wild type and the complemented strains confirmed the presence of numerous fimbriae all over the surface (Fig. 2c,e) while the ∆*fimA* strain showed no fimbriae on its cell surface (Fig. 2d). From enlarged AFM images of the wild type, ∆*fimA* and *∆fimA::pQE30fimA UPEC* strains in the height mode (Fig. 2c’–e’) and the height and diameter of fimbriae (Fig. 2c”–e”) in the AFM images ensured the change in the cell surface and fimbriae. The diameter of fimbria was found to be approximately 5–7 nm and several micrometers in length, which is consistent with a previous report^30^. To assess the interaction of MCSNP with fimbriae, AFM images of *UPEC* were analyzed after treating the bacteria with 1 μg/mL MCSNP. The AFM analysis confirmed the binding of MCSNPs on fimbriae fibrils (Fig. 2f and inset). From the characterization of ∆*fimA UPEC* strain, we were able to confirm that the mutant strain has no fimbria, and thus it can be predicted that the trapping efficiency of MCSNP on the mutant strain expected to be lower than wild type strain. To verify this prediction, wild type *UPEC, ∆fimA,* and *∆fimA* complemented strains were subjected to MCSNP-mediated magnetic-trapping. As expected, the trapping efficiency of the mutant was reduced to 45% of the wild-type *UPEC* strain efficiency (Fig. 2g), but the complemented strain recovered the trapping efficiency.

**Figure 3:**
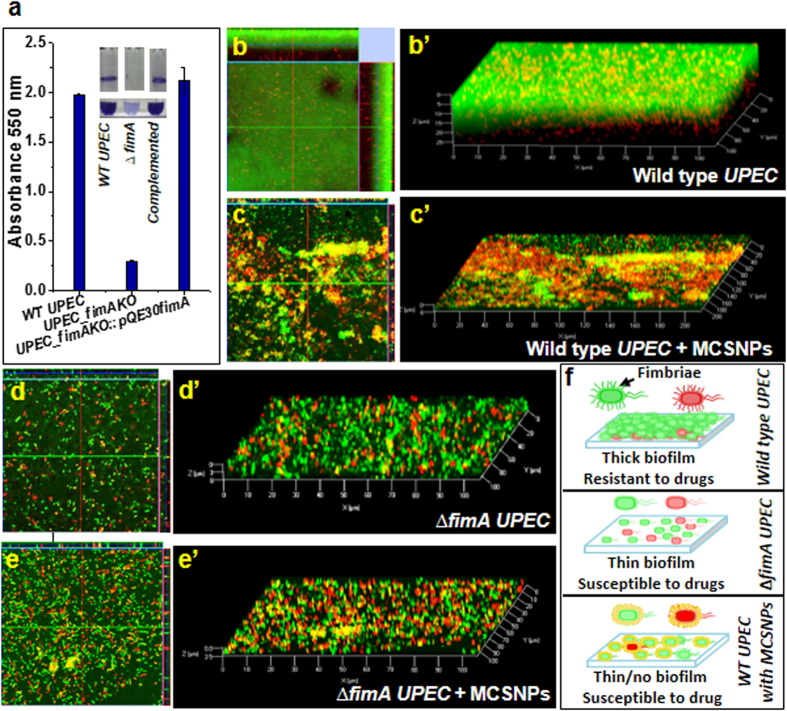
Inhibition of *UPEC* biofilm formation by MCSNPs. **(a)** Biofilm formation by wild type *UPEC*, mutant (∆*fimAΩkm*^*r*^), and complemented (∆*fimAΩkm*^*r*^*::pQE30fimA*) strains is quantitatively analyzed by staining with crystal violet and measuring the absorbance 550 nm. Insets display the biofilm in tubes. Top view and Z-stack images of biofilm formed by wild type *UPEC* without (**b**, **b’**) and with MCSNP (**c**, **c’**), and ∆*fimAΩkm*^*r*^ strains without (**d**, **d’**) and with MCSNP (**e**,** e’**). The samples were stained by fluorescein diacetate and propidium iodide before examination with confocal laser scanning microscopy. The biofilm thicknesses in **b’–e’** were calculated to be 28, 6, 6, and 3.5 μM, respectively. (**f**) Schematic diagram showing the role of type I fimbriae in biofilm formation (top), inhibition of biofilm formation by MCSNPs (bottom), and failure of biofilm formation in the ∆*fimA* strains (middle).

Since fimbriae are known to be main players in biofilm formation^31^,^32^, we further characterized the phenotype of the ∆*fimA* strain by investigating its biofilm formation on polypropylene substrate using the crystal violet staining. The ∆*fimA* strain showed only 12.5% biofilm formation capacity compared to the wild type and complemented strains (Fig. 3a), confirming fimbriae have a primary role in *UPEC* biofilm formation as reported earlier in the other commensal *E. coli* strain^31^. Accordingly, we hypothesized that bacteria treated with MCSNPs show reduced biofilm formation capacity since MCSNP binding to fimbriae possibly blocks fimbriae-surface and fimbriae-fimbriae interactions, which are the key steps and prerequisites of biofilm formation. The fluorescein diacetate (FDA) and propidium iodide (PI) stained biofilms of wild type and ∆*fimA UPEC* without or with MCSNP were analyzed by confocal microscopy (Fig. 3b–e) and biofilm thickness was analyzed by z-stack image where one stack is equivalent to 1 μm (Fig. 3b’–e’). As expected, wild type *UPEC* produced the thickest biofilm, up to 28 μm, when MCSNP was absent (Fig. 3b–b’). However, treatment of MCSNP to the wild type *UPEC* reduced the biofilm thickness to 6 μm (Fig. 3c–c’), which was similar to that of ∆*fimA* strain (Fig. 3d–d’). The treatment of MCSNPs to the ∆*fimA* strain further reduced the biofilm thickness of ∆*fimA* strain to 3.5 μm (Fig. 3e–e’). These results suggest that MCSNP can also block biofilm formation mediated by other factors in addition to fimbriae. MCSNP treatment resulted in a 78.6% reduction in biofilm thickness of wild type *UPEC.* Significant reduction in biofilm thickness owing to the treatment with MCSNP is likely achieved due to the unavailability of free fimbriae required for the interaction with a surface for bacterial attachment. Taken together, these results indicate that the MCSNP-trapped wild type *UPEC* possesses the cell surface analogy with the ∆*fimA* strain by having fimbriae buried inside MCSNP. Accordingly, MCSNP can effectively block biofilm formation by inhibiting cell-cell interactions within the community. The schematic diagram showing biofilm formation by wild type *UPEC* and ∆*fimA* with and without MCSNP is depicted in Fig. 3f where the wild type strain formed a thicker biofilm and consequently creates a stronger physical barrier to drug entry than *∆fimA* or MCSNP-complexed WT strain.

The binding mode and functionality of MCSNP as a biofilm inhibitor of Gram-positive bacteria were also investigated to broaden its environmental applicability. For this purpose, a representative Gram-positive methicillin resistant *Staphylococcus aureus* strain USA300 FPR3757 (hereafter referred as *MRSA*) was used. Although pili/fimbriae are absent in most of the Gram-positive bacteria, the overall surface charge remains negative due to the abundance of long chains of lipoteichoic acid (LTA)^33^, which play a crucial role in biofilm formation^34^,^35^. By applying the same analyses as *UPEC* to *MRSA*, we found that MCSNP binds on the bacterial surface (Supplementary Figure 5a) and capturing efficiency for *MRSA* is 95.3% (Supplementary Figure 5b). From these analyses, it is expected that MCSNP binding to the LTA can block biofilm formation. When biofilm formation was investigated using *MRSA* without and with MCSNP treatment at various concentrations (100, 200, and 500 μg/mL), it was confirmed that biofilm inhibition is proportional to the concentration of MCSNP, and complete inhibition was achieved at 1 mg/mL (Supplementary Figure S6A–C). It has been reported that the minimal inhibitory concentration (MIC) of drugs or antibiotics can increase up to 1000-fold by biofilm formation and host cells get more damage, and thus antibiotics are not effective on MDRB^11^,^36^,^37^,^38^. In these aspects, it will be advantageous to use MCSNPs for the treatment of biofilm of the pathogenic MDRB since it is expected that MCSNPs can significantly lessen biofilm formation and thereby drug resistance.

**Figure 4:**
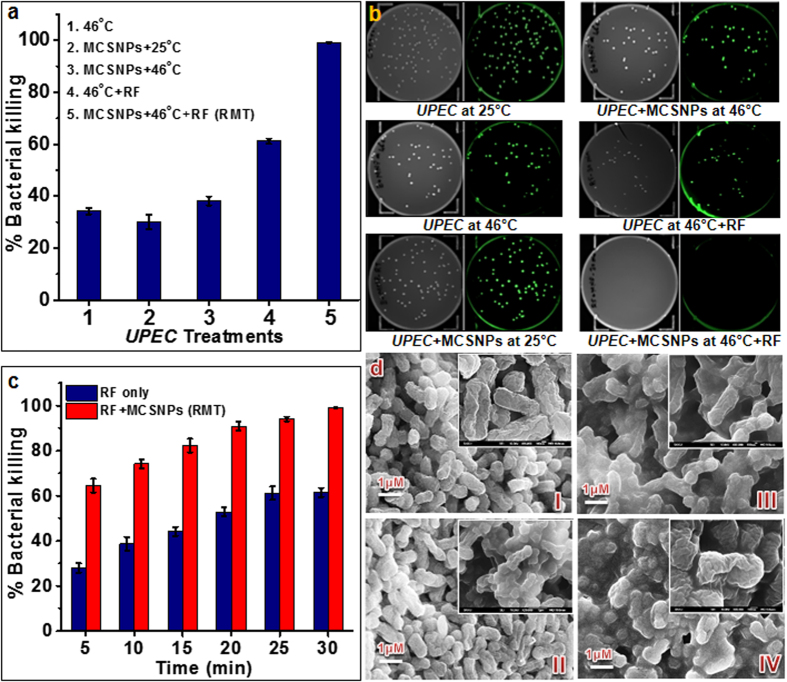
Antibacterial efficiency of radiofrequency (RF) alone or when coupled to magnetic nanoparticle treatment (RMT). **(a)** Effects of various antibacterial treatments on *UPEC* death. Radiofrequency (RF, 400 kHz) coupled to MCSNP treatment (RMT) showed 100% killing within 30 min at 45 °C. The antibacterial efficacy of the other conditions indicated in the figure legends was much lower. **(b)** Antibacterial efficiencies of various treatments against EGFP expressing *UPEC,* as measured by CFU. **(c)** Effect of RF time on antibacterial activity against free-living (RF only) or MCSNP-trapped (RMT) *UPEC*. **(d)** Scanning electron microscopic (SEM) microphotographs of *UPEC* treated at various conditions for 30 min: control *UPEC* cells at (I) 25 °C and (II) 46 °C, (III) *UPEC* treated with RF only at 46 °C, and (IV) *UPEC* trapped with MCSNPs and treated by RF at 46 °C (RMT).

The antibacterial or bacteriostatic properties of various nanoparticles such as silver, zinc oxide, titanium oxide, copper, aluminum, and iron have been reported and reviewed recently^12^. However, since not many studies have followed the protocol of the Clinical and Laboratory Standard Institute (CSLI)^39^, their antibacterial or bacteriostatic potential cannot be evaluated properly. Furthermore, it has been also reported that metallic Ag-NPs inherently possess negligible antibacterial activity and their broad range of toxicity to cells are mainly attributed to the release of silver ions^40^. Therefore, NPs themselves seem to not be practically useful as potent antibacterial agents. As an alternative approach, radiofrequency radiation (RFR) at 2450 MHz has been reported to kill *Bacillus anthracis* due to ROS generation^41^. Based on these results, we hypothesized that the combination of NPs and RF may enhance the antibacterial effect as it has been known that the coupling of NPs with electromagnetic energy can be used for a wide range of medical applications^21^,^22^,^24^. To test this possibility, we introduced radiofrequency coupled magnetic nanoparticle treatment (RMT) by exposing magnetic nanoparticles to radiofrequency (RF) current using a CET system. To the best of our knowledge, the coupling of radiofrequency with NPs for antibacterial purposes has not been explored. In this setting, solutions containing bacteria were exposed to radiofrequency in the presence of MCSNPs (RMT group) or the absence of MCSNPs (RF group). The quantitative and qualitative assessment of killing efficiencies of RMT *versus* RF were estimated by counting the colony forming units (CFU) and live/dead staining, respectively. Bacterial death was observed after the microbes were exposed to the heat released from the NPs during RF conditions. However, to examine the sole effect of RF on bacterial killing, temperature was maintained at a constant value by adjusting the radiofrequency power. As a control, the conditions for 100% bacterial killing were optimized by testing varying concentrations of MCSNPs, exposure times, and incubation temperatures and the following optimal conditions were determined: 1 × 10^8^ *UPEC* cells were treated with 1 mg/mL MCSNPs (10 pg per cell equivalent) and exposed to 0.4 MHz RF at 46 °C for 30 min (Fig. 4a). Then, the killing efficiency of the control condition was compared at 25 °C (RT) and 46 °C in the absence or presence of MCSNPs. *UPEC* incubated at 46 °C for 30 min showed 37% killing, while MCSNP-trapped bacteria at 25 °C and at 46 °C showed about 30% and 40% killing, respectively (Fig. 4a,b). Free bacteria without MCSNPs with RF at 46 °C showed 60% killing while RMT killed 100% bacteria within 30 min at 46 °C (Fig. 4a,b). The higher killing efficiency of RMT was also examined by live/dead staining of *UPEC* and microscopic observation, and there was no indication of live bacteria observed after RMT treatment (Supplementary Figure S7). To estimate the effectiveness of RF coupled to MCSNPs, time dependent killing was monitored during RF *versus* RMT. The bacterial killing efficiency in the initial 5 min of RF and RMT were found to be 28% and 65%, respectively. Bacterial killing efficiency increased with exposure time and 60% and 100% killing were achieved within 30 min for RF and RMT treatments, respectively (Fig. 4c). To perceive the phenotypic alteration during RF *versus* RMT along with other controls *i.e. UPEC* at 25 °C and 46 °C, the exposed cells were subjected to scanning electron microscopy (SEM). SEM images showed alteration in bacterial cell surfaces during RMT (Fig. 4d–IV) compared to 25 °C, 46 °C, and RF controls (Fig. 4d I-III) where the RMT treated bacteria showed changes in cell surface morphology (Fig. 4d–IV) compared to the corresponding controls.

Under our experimental conditions, maximum killing efficiency was obtained when bacteria were treated with MCSNPs in combination with RF exposure (RMT), while heat only (46 °C), MCSNPs only, MCSNPs with heat, or RF with heat resulted in suboptimal killing activity (Fig. 4a,b). Generally, it is considered that coupling RF or other electromagnetic waves with NPs in living systems has the advantage of generating heat, which possesses immense killing capability in cells depending on the experimental conditions. However, current results revealed that bacterial killing was not solely caused by heat energy since killing efficiency of control conditions except RMT has not achieved 100%, despite maintaining the temperature at 46 °C (Fig. 4a). These results suggest the existence of additional lethal factors or mechanisms in RMT.

**Figure 5:**
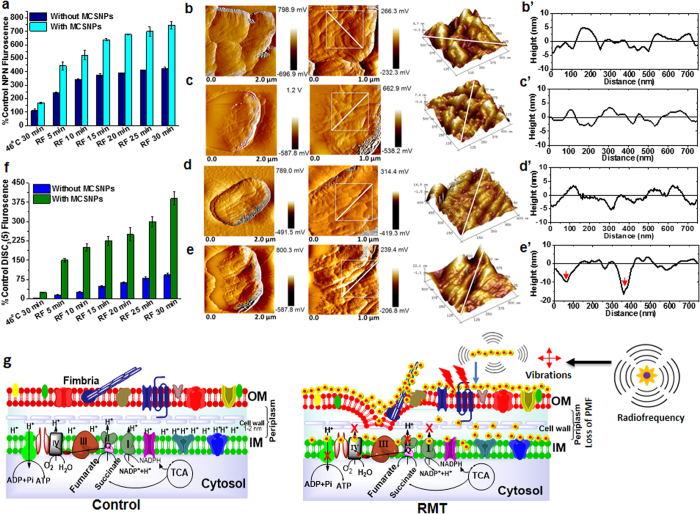
Membrane perturbation of *UPEC* by RMT. **(a)** Outer cell membrane permeability of *UPEC* by RF and RMT treatments. **(b–e’)** AFM amplitude mode images of *UPEC* cell surfaces after treatment at various conditions: (**b–e**) cell morphology and (**b’–e’**) outer membrane topography analyses of *UPEC* incubated at 46 °C without MSCNPs (**b**,**b’**), and with MCSNPs (**c**,**c’**), *UPEC* treated with RF at 46 °C without MCSNPs (**d**,**d’**), and with MCSNPs at 46 °C (RMT, **e**,**e’**). Note that carter-type pits with 10–17 nm depths towards the inner membranes were observed after RMT treatment (**e’**). (**f**) Inner membrane (IM) potential of *UPEC* after RF or RMT treatment at 46 °C. (**g**) Schematic diagrams showing the *UPEC* membrane and membrane associated electron transport chain in control cells (left) and cells after RMT treatment (right). Outer membranes were damaged and crater-like pits were formed by ROS, heat, and mechanical vibration from NPs after RF application. Consequently, loss of inner membrane potential and dysfunction of membrane associated complexes followed. Eventually, these factors induce bacterial cell death.

Recently, it was reported that upon RF exposure, iron oxide magnetic nanoparticles attached to the liposome can deflect and perturb the membrane, acting as mechanical transducers to transfer the energy of radiofrequency to the liposome^42^. Since MCSNP can closely pack on the bacterial surface (Fig. 2b), it is tempting to hypothesize that the MNP can mechanically perturb the cell membrane under a RF field. This possibility was further investigated by measuring the outer membrane (OM) permeability of bacteria bound to MCSNPs using hydrophobic 1-N-phenylnaphthylamine (NPN) that shows fluorescence when it attaches to the phospholipid layer after passing through the outer membrane. The NPN fluorescence from the RMT samples was 5 fold higher than control (*UPE*C incubated at 46 °C for 30 min), while the fluorescence from the RF-treated samples was just 3 fold higher than control (Fig. 5a), suggesting a greater loss of the permeability barrier during RMT. To further examine the membrane permeability in detail, bacterial cell surfaces were visualized by AFM after removal of MCSNP using PBS buffer (Fig. 5b–e’). Control *UPEC* cells incubated at 46 °C without MCSNP, or exposed to RF at 46 °C, showed no noticeable alteration in their surface (XY plane at Fig. 5b–d and Z-axis at Fig. 5b’–d’) and were found to be comparable to the cell surface treated at 25 °C without or with MCSNP (Supplementary Figure S8A,B). However, *UPEC* strains showed crater type pits in their OM with increased roughness (~10–17 nm deep) under RMT (Fig. 5e–e’). To get an in-depth insight into the bioenergetics of the cells upon perturbation of the OM barrier, the inner membrane (IM) potential (∆ψ) was measured using lipophilic DiSC_3_(5), a potentiometric fluorescent probe representing the membrane potential by emitting fluorescence when it is released from the membrane upon collapse of the transmembrane potential due to membrane depolarization^43^. As expected, RF combined with MCSNP drastically lost IM potential compared to RF treatment alone at 46 °C (Fig. 5f). Taken together, these results suggest that membrane-bound MCSNP can damage OM and the IM potential under RF, which causes bacterial death.

The RF-based heating of various NPs has been reported but its mechanism is often a matter of debate^44^,^45^. The heating mechanism of superparamagnetic iron oxide NP under electromagnetic fields is proposed to be related to the Brownian and Néel’s spin relaxations and heating through hysteresis loss^46^. Recently, the use of electric fields with intermediate ranges of radiofrequency (100–300 kHz) are being studied for the treatment of tumor, in which rapidly dividing cells are killed by dielectrophoretic force^47^. In addition, it is proposed that RF causes vibrational motion of NPs due to their magnetic moment^48^. Consistently, mechanical membrane deflection by iron oxide nanochains under radiofrequency has been demonstrated using liposomal membranes for targeted drug delivery^42^. Considering these observations, the plausible reasons for efficient antibacterial activity during RMT can be proposed as being due to radiofrequency-mediated heating and efficient heat transfer to the cell membrane by MCSNP through the direct contact of NPs to the cell membrane, and/or by vibration of MCSNPs on bacterial membranes by RF. Consequently, the loss of their permeability barrier and inner membrane potential is likely to be caused by a combination of the two effects. In addition, it is also reasonable to propose that dielectrophoretic forces under electric field and intermediate range RF exert physical forces on *UPEC* cells as reported in case of cancer cell replication^47^. Taken together, the aforementioned biophysical parameters adversely affect *UPEC* membranes and result in dysfunction of membrane associated complexes and their related vital biological activities (Fig. 5g). It is noteworthy that the bacterial membrane is the key cellular component where various vital biochemical and bioenergetics processes takes place. Therefore, loss of bacterial membrane potential would cause the collapse of the physiological processes associated with the membrane, such as the electron transport chain, proton motive force (PMF), and ATP production, which results in efficient antibacterial activity. Therefore, enhanced bacterial killing under RMT is likely attributed to RF-mediated physical perturbation of cell membranes and bacterial membrane dysfunction.

The RMT physical antibacterial therapy possesses several advantages over chemical antibiotics in the treatment of superbugs because (**a**) there is no chance to develop intracellular bacterial community (IBC) for secondary infections which is common in *UPEC* infection during antibiotic treatment, (**b**) there is no chance for adaptive evolution or natural selection of antibiotic resistant bacteria, and (**c**) this treatment also avoids antibiotic-induced biofilm formation, a survival strategy of bacteria against antibiotic treatment. Furthermore, RMT is free from safety issues since iron oxide has been clinically used as an MRI contrasting agent, and low energy RF is also widely used for many medical and cosmetic purposes. The mechanistic understanding of RMT and its exceptional capability to kill MDR bacteria has an enormous potential in medical applications, and thus holds great promise for future translational nanomedicine for treating infectious diseases that cannot be cured by conventional antibiotic therapy. For example, RMT can be used for the treatment of UTI patients infected by the colistin resistant *E. coli* strain SHP45 which possesses and disseminates *mcr-1*, a plasmid-mediated colistin resistance gene^49^. In addition, it also has the potential to be used for remedying environmental contamination with MDRB. Therefore, this study could be a milestone as well as a cornerstone for the physical treatment of bacteria, and our new method may serve as an antibacterial alternative to chemical antibiotics.

## Methods

### Synthesis of Fe_3_O_4_ magnetic nanoparticle (MNP) core

Iron (III) acetylacetonate, Fe(acac)_3_ (1.765 g, 0.1 M) was dissolved in a water/ethanol (v/v 1:1) mixture (50 mL), and the solution was purged with nitrogen gas for one hour. Ice cold sodium borohydride (NaBH_4_, 1.8915 g, 1 M) was added with magnetic stirring at 1200 rpm under a nitrogen atmosphere overnight. At this stage, evolution of hydrogen (H_2_) gas was observed and the solution color was changed rapidly from red to brown, with the black color indicating formation of the Fe_3_O_4_ nanoparticle core. The reaction mixture was stirred at 1200 rpm for an additional hour in a nitrogen atmosphere. The formed superparamagnetic iron oxide nanoparticles were magnetically separated, washed repeatedly with ethanol and water until complete removal of surface absorbed NaBH_4_, and then dried under nitrogen at room temperature.

### Synthesis of amine-functionalized Fe_3_O_4_@SiO_2-_NH_2_ core-shell NPs

The amine-functionalized Fe_3_O_4_@SiO_2_ core-shell nanoparticles were synthesized through a one-step method^50^. Iron oxide core (Fe_3_O_4_) NPs (50 mg) was dispersed in 25 mL 80% ethanol by ultrasonication. One mL of tetraethyl orthosilicate (TEOS) was added to the mixture solution. After 4 h of stirring, 0.5 mL of 25% ammonia solution was added dropwise to initiate TEOS hydrolysis, and the reaction proceeded with stirring at room temperature for 24 h. After 24 h, 0.4 mL of (3-aminopropyl) triethoxysilane (APTES) was added to the solution and the mixture was stirred for an additional 24 h at 60 °C. The resultant Fe_3_O_4_@SiO_2_ core-shell NPs were separated from the solution *via* centrifugation, purified by washing with water and ethanol several times, and then were dried under a vacuum for 12 h.

### Characterization of magnetic core-shell Fe_3_O_4_@SiO_2-_NH_2_ NPs (MCSNPs)

The size and morphology of magnetic core-shell nanoparticles (Fe_3_O_4_@SiO_2_–NH_2_, MCSNPs) were observed under a high resolution transmission electron microscope (HRTEM) (Cs_corrected/EDS/EELS model JEM ARM 200F, JEOL, USA). X-ray photoelectron spectroscopy (XPS) spectra were collected on an ESCALAB 250 multi-technique X-ray photoelectron spectrometer (Thermo Scientific, UK) using a monochromatic Al Kα x-ray source (hυ = 1486.6 eV). The magnetization measurements were performed on a Quantum Design SQUID magnetometer to investigate the magnetization properties of the nanoparticles. Particle size distribution and zeta potentials were measured with a PSS-NICOMP-380 ZLS (USA) particle sizing system.

### Bacterial growth and culture conditions

All the primers, plasmids, and recombinant bacterial strains used in this study are presented in Tables S1 and S2. Strains of *Escherichia coli (E. coli* DH5α, and uropathogenic *E. coli* CFT073, *UPEC*) were grown in Luria Bertani (LB) broth medium or LB-agar (1.5% agar) plates at 37 °C. The broth cultures were grown under orbital shaking at 200 rpm while the strains on plates were grown in a 37 °C incubator overnight. Strains of methicillin-resistant *Staphylococcus aureus (MRSA*) were grown in tryptic soy broth (TSB) or brain heart infusion (BHI) media under orbital shaking at 200 rpm or on tryptic soy agar plates (TSA, 1.5% agar) at 37 °C. Cell growth was monitored by measuring the optical density at 600 nm (OD_600_). Appropriate amounts of antibiotics such as ampicillin (Amp, 100 μg/mL), kanamycin (Km, 50 μg/mL), chloramphenicol (Cm, 33 μg/mL), or streptomycin (Sm, 50 μg/mL) were added to the cultures of recombinant *E. coli* strains. For TSB culture and TSA plates, 12.5 and 25 μg/mL chloramphenicol were used, respectively, for culturing the recombinant *S. aureus* USA300 FPR3757 strain stably expressing *luxBADCE* genes (*MRSA’*) integrated into the genome.

### Recombinant DNA techniques

The molecular biology experiments conducted followed the standard protocols appearing in the laboratory manual book^51^ unless otherwise specified, and molecular biology kits were used according to the manufacturer’s protocols. Plasmid DNA was isolated using a plasmid DNA isolation kit (Intron Biotech, Korea). Genes were amplified using high fidelity Taq polymerase following the standard PCR protocol. The PCR amplified DNA fragments or genes were subjected to gel electrophoresis and purified using a gel purification kit (Intron Biotech, Korea). The DNA fragments were digested using restriction endonucleases (New England Biolab, USA), then were ligated and cloned into the corresponding vectors using ligase (Takara, Japan). The sequence identity of PCR amplified gene/mutant alleles was verified by DNA sequencing.

### Expression of enhanced green fluorescent protein (EGFP) in wild type *E. coli* CFT073 (*UPEC*) strains

Colony forming units (CFU) of *UPEC* were measured using recombinant UPEC strain expressing EGFP. The EGFP expressing recombinant UPEC strain was created by electroporation of *pluxI-EGFP* plasmids (streptomycin resistance, *sm*^*r*^). Briefly, electrocompetent cells were prepared by washing cells growing in log phase with 10% glycerol at 4 °C as previously described^52^. The plasmid was electroporated using a GenePulserXcell^TM^ (BIORAD, USA) at 2000 V with a gap between electrodes of 1 mm, 200 Ω resistance, and 25 μF capacitance. The electroporated cells were allowed to recover in LB broth at 37 °C for 1 h. The recovered cells were plated on 1.5% LB-agar plates supplemented with streptomycin and incubated at 37 °C overnight. The EGFP fluorescence of cultures was then measured by using a spectrofluorometer (JASCO, Japan) or microtiter plate reader (BioTek, USA).

### Deletion of *fimA*, a gene encoding fimbriae, and complementation of ∆*fimA* knockout strain

The *fimA* gene (606 bp) was deleted and replaced by a kanamycin resistance cassette (*km*^*r*^) using a red recombinase system (Supplementary Figure 4A) as previously described^53^. Briefly, the red recombinase encoding plasmid pKD46 was electroporated into wild type *E. coli* CFT073 and the colonies were selected on LB agar plates containing ampicillin grown at 30 °C. The lambda red recombinase enzyme was induced by adding arabinose and growing bacteria at 30 °C. Primer pairs (*FimAFRT KO_fwd* and *rev,* Table S1) containing FRT sites with 40 bp flanking regions of *fimA* gene were used for PCR amplification of the kanamycin cassette from pKD4 plasmid. The linear DNA was gel purified and treated with DpnI enzyme for 2 h at 37 °C. The mutant allele was ethanol precipitated and washed with 70% ethanol. The purified linear DNA mutant allele (500 ng) was electroporated into red recombinase expressing *UPEC* electrocompetent cells and the putative mutant colonies were selected on kanamycin-containing plates. The kanamycin resistant colonies were verified for the *fimA* deletion and insertion of kanamycin cassette using PCR (Supplementary Figure 4B). The red recombinase plasmid was cured by growing the mutant strain at 42 °C. The *fimA* gene was PCR amplified using the primer pairs *fimAfwd* and *fimArev* primers shown in Table S1. The *fimA* gene was cloned into *EcoRI* and *HindIII* sites of *pQE30* vector to achieve plasmid *pQE30fimA* to complement the *∆fimAΩkm*^*r*^ knockout strain. The plasmid *pQE30fimA* was elctroporated into ∆*fimAΩkm*^*r*^ knockout for complementation to achieve ∆*fimAΩkm*^*r*^::*pQE30fimA* strain and colonies were selected on LB agar plates containing kanamycin and chloramphenicol (Supplementary Figure 4B).

### Atomic force microscopy (AFM) imaging of bacterial strains

The glass substrate (Eagle XG, Korea) for AFM imaging was prepared as previously described^54^. Briefly, glass slides were immersed in a solution of ethanol and hydrochloric acid (v/v 70/1) overnight and washed with deionized water. This procedure was repeated twice before using the glass substrate for AFM imaging. The bacterial strains (wild type *E. coli* CFT073, knock-out *∆fimAΩkm*^*r*^, and complemented strain *∆fimAΩkm*^*r*^::pQE30*fimA*) were fixed with 2.5% glutaraldehyde for 2 h as previously described^54^. Ten μL of cell solutions with an OD_600_ of 0.1 were drop cast on a 5 × 5 × 0.5 mm glass substrate and were allowed to air-dry at room temperature for 30 minutes. The glass substrate was mounted on a metal puck. AFM images were obtained at room temperature using a Multimode Nanoscope (Veeco Inc., USA) in air tapping mode with silicon cantilevers having a spring constant of 42 N/m. Height, amplitude, and phase images were obtained simultaneously. Initially, 50 × 50 μm^2^ scan size images were captured to locate the bacterial cells, followed by a gradual decrease in scan size to the point where fimbriae were clearly visible.

### Generation of bioluminescent strains of *S. aureus* USA300 (*MRSA’*) and *E. coli* CFT073 (*UPEC’*)

The plasmid *pGEN-luxCDABE* (a generous gift from Prof. Harry L. T. Mobley, University of Michigan Medical School, Ann Arbor, MI, USA)^55^ was electroporated into wild type *UPEC* to create the bioluminescent strain (*UPEC’*), where the *luxCDABE* gene was constitutively expressed under synthetic promoter em7. The bioluminescent *S. aureus* USA300 FPR3757 (*MRSA’*) strain was created by transduction as previously described^56^. Briefly, the strain SAP140 of RN4220 harboring pRP1195 plasmid^57^ (a generous gift from Prof. Scott Stibitz, Food and Drug Administration, Bethesda, Maryland, USA) was grown in TSB media supplemented with chloramphenicol and 5 mM calcium chloride up to an OD_600_ of 0.5. The phage Ø85 particle was added and growth was allowed until visible cell lysis. Virus particles containing pRP1195 were prepared by passing the overnight grown culture through a 0.22 μM filter. Plasmid pRP1195 was transferred to the *S. aureus* USA300 FPR3757 strain by transduction as previously described^56^. The recombinant strain was selected on TSA plates supplemented with 40 mM sodium citrate and 25 μg/mL chloramphenicol. A single colony was streaked on TSA plates containing chloramphenicol repeatedly to purify the colony. The plate-purified single colony was grown at 43 °C to integrate the plasmid into the genome for stable expression of bioluminescence. The culture grown at 43 °C was plated on TSA plates containing chloramphenicol to achieve bioluminescent *MRSA’* strains.

### Bacterial trapping efficiencies using MCSNP

Bacterial cell (*S. aureus* USA300, *MRSA* and *E. coli* CFT073, *UPEC*) numbers were adjusted to an OD_600_ of 1 (1 × 10^8^ cells/mL for *UPEC* and *UPEC’*, and 1 × 10^9^ cells/mL for *S. aureus* USA300, *MRSA, MRSA’*) in phosphate buffered saline (PBS, pH 7.4). Varying concentrations of MCSNPs were added into bacterial suspensions while maintaining a final volume of 1 mL. After 10 minutes of incubation, an external magnet was employed for magnetic separation of MCSNP-trapped bacterial cells. The supernatant was then carefully pipetted out into cuvette to measure OD_600_ for wild type cells or luminescence (*via* Luminometer) for bioluminescent strains (Table S2). The percent efficiencies of magnetic capturing were estimated as an OD_600_ and/or bioluminescence ratio between the MCSNP-captured bacteria and control bacteria that was incubated without magnetic capture.

### Biofilm formation and its qualitative and quantitative study

The effect of magnetic capture of bacteria on biofilm formation was studied by using a microtiter dish/culture tube biofilm formation assay protocol^58^. Briefly, wild type *UPEC* or *MRSA* strains were grown overnight and diluted 1:100 in fresh medium for the biofilm assay. MCSNPs at varying concentrations were added to the microtiter wells/tube/confocal slides containing diluted cell suspensions and were incubated for 24 h at 37 °C to allow for biofilm formation. After 24 h of incubation, the cell suspension was removed carefully and replaced by fresh media and incubated for an additional 24 h. After incubation, the plate was gently submerged in a small tub of autoclaved MilliQ water and water was removed by gentle flipping. This process was repeated once again. For quantitative analysis of biofilm, 0.1% aqueous crystal violet solution was added to each well of the microtiter plate/tubes. The microtiter plate/tubes were incubated at room temperature for 20 min. Plates were rinsed with water by submerging in a tub of water and plates were dried overnight. Aqueous acetic acid (30%) was added to each well of the microtiter plate to solubilize the crystal violet-stained biofilm by incubating at room temperature for 20 min. The solution was transferred to a new flat bottomed microtiter dish or cuvette; and the absorbance was measured at 550 nm using 30% acetic acid as a blank. For qualitative analysis of biofilm, the confocal slide was submerged in water to remove any free cells from the cell suspension. Then 100 μL PBS along with 10 μL fluorescein diacetate (FDA) only or with propidium iodide (PI) were added in each well of the plate and was incubated for 10 min. Plates were washed twice to remove FDA and PI that were nonspecifically bound to the surface. The surface and depth of biofilm was observed and measured, respectively, under a confocal microscope (at 40X or 20X magnification Zeiss LSM 510 Meta). Red and green fluorescent cells were observed by the optimal excitation and emission wavelengths: PI (λ_excitation_ = 535 nm, λ_emission_ = 617 nm) and FDA (λ_excitation_ = 495 nm, λ_emission_ = 519 nm).

### Killing efficiencies of *UPEC* by radiofrequency (RF) *versus* radiofrequency coupled magnetic nanoparticle treatment (RMT)

The initial cell number of EGFP expressing *UPEC* was maintained at an OD_600_ of 1 for radiofrequency (RF) experiments. The RF treatment was applied to either free bacteria (without MCSNPs) or with 1 mg/mL MCSNPs (RMT) in PBS to estimate the RF *versus* RMT killing in 10 mL PBS buffer (total, 1 × 10^8^ *UPEC* cells). The control cells at 25 °C and 46 °C were incubated with or without MCSNPs to evaluate the individual effects of temperature and MCSNPs on antibacterial activity. The RF was generated by a capacitive electric transfer (CET) system as previously reported^24^. Briefly, two insulated metal plates (2 cm diameter, 8 cm gap) connected to the RF generator acted as active and return electrodes, respectively. A glass cylinder of 8 cm in length and 2 cm in diameter works as an incubation chamber. Ten mL of bacterial solution in PBS (pH 7.4) was placed in the incubation chamber for RF treatment. Frequency and output power were measured using an oscilloscope TDS 210 (Tektronix, Inc., Beaverton, OR, USA). RF with a frequency of 0.4 MHz and power in the range of 30–40 W was applied for the RF treatment. The temperature of the incubation chamber containing the bacterial sample was monitored using a fiber optic temperature sensor system with an accuracy of ±0.1 °C. After time and temperature dependent exposure of RF on without or with MCSNPs trapped bacteria, samples were diluted 6 fold and the killing percentage was determined by counting the colony forming units (CFU).

### Reactive oxygen species (ROS) measurement

Total levels of reactive oxygen species were measured using 2′,7′dichlorodihydrofluorescein diacetate (DCFH-DA) fluorescent dye to determine the generation of free radicals during RMT on *UPEC* along with other controls^59^. RMT exposed, RF treated, and RF-untreated control cells at 25 °C and 46 °C with or without MCSNPs samples were washed three times in PBS and equal cell numbers of all samples including control were adjusted to an OD_600_ equivalent of 0.1. The bacterial samples were stained with 10 μM ROS-specific fluorescent dye DCFH-DA for 30 min in the dark at the room temperature and were washed with PBS once. Fluorescence was measured using a microplate reader with excitation at 488 nm and emission at 525 nm (BioTek, USA). The total ROS level is presented as a percent relative to the control at room temperature with or without MCSNPs. All the samples were measured in triplicate and data is presented as mean ± SD.

### Assessment of bacterial membrane biophysical alteration

#### Outer membrane permeability

For the outer membrane permeability assay, cells were washed once in 5 mM HEPES buffer (pH 7.2) and resuspended into the same buffer. The cell numbers were adjusted to an OD_600_ of 0.1. One mL of this cell suspension was incubated with 10 μL of 1-N-phenylnaphthylamine (NPN) solution (10 mM) in the dark for 30 min at room temperature, washed twice with PBS, and fluorescence intensity at 420 nm was measured after excitation at 350 nm. The total NPN fluorescence is presented as a percent with respect to control at 25 °C incubated with or without MCSNPs. All readings were measured in triplicates and data is presented as mean ± SD.

#### Inner membrane potential

The inner membrane potential was measured using similar procedures to the outer membrane assay. Cell numbers were adjusted in HEPES/glucose buffer (5 mM HEPES, 5 mM glucose, pH 7.0) to an OD_600_ equivalent to 0.1. One mL of this bacterial cell solution was incubated with 10 μL of 10 mM 3,3′-dipropylthiadicarbocyanine iodide (DiSC_3_(5)) solution in dark at the room temperature for 1 h. All samples were equilibrated with 100 mM KCl for 1 h, and fluorescence at 670 nm was measured after excitation at 622 nm.

#### Preparation of bacterial samples for scanning electron microscopy (SEM)

RMT, RF, and control *UPEC* cells at 25 °C and 46 °C were collected by centrifugation at 5000 rpm for 2 min and were washed twice with PBS. The cells were fixed in 2.5% glutaraldehyde overnight at 4 °C. The bacterial cells were washed with PBS three times after fixation. The cells were dehydrated using ethanol gradient of 10, 20, 40, 80% and finally were resuspended in 100% ethanol. Finally, samples were put on the silicon grid and observed under SEM.

#### Live/dead staining

The RMT, RF, and control *UPEC* cells at 25 °C and 46 °C were washed once in PBS. The OD_600_ of all samples was adjusted to 0.1 (1 × 10^7^ cells). For live/dead staining, 900 μL cell samples were stained with 100 μL FDA and PI for 10 min and were washed twice using PBS to remove unbound dye. Ten μL stained cells were drop cast on several glass slides for each sample, covered by a coverslip, and sealed with nail polish. Fluorescence images were taken under a confocal microscope (40X magnification Zeiss LSM 510 Meta). Red and green fluorescent cells were observed at the appropriate excitation and emission wavelengths, PI (λ_excitation_ = 535 nm, λ_emission_ = 617 nm) and FDA (λ_excitation_ = 495 nm, λ_emission_ = 519 nm), and detected with a band-pass filter, with the final images generated by superimposing the red and green images.

## Additional Information

**How to cite this article**: Chaurasia, A. K. *et al*. Coupling of radiofrequency with magnetic nanoparticles treatment as an alternative physical antibacterial strategy against multiple drug resistant bacteria. *Sci. Rep.* **6**, 33662; doi: 10.1038/srep33662 (2016).

## Supplementary Material

Supplementary Information
