# Supplementary material for: Coupling of radiofrequency with magnetic nanoparticles treatment as an alternative physical antibacterial strategy against multiple drug resistant bacteria
Source: Sci Rep. 2016 Sep 27;6:33662. doi: 10.1038/srep33662 (PMC5037373; doi:10.1038/srep33662)
Supplement: Supplementary Information [file srep33662-s1.pdf]

ARTICLE TITLE

Coupling of radiofrequency with magnetic nanoparticles treatment as an alternative physical antibacterial strategy against multiple drug resistant bacteria

RUNNING TITLE

Radiofrequency-magnetic nanoparticles based antibacterial strategy

Akhilesh K. Chaurasia[¶1], Nanasaheb D. Thorat[¶1], Anshula Tandon[2,3], Jin-Hahn Kim[1], Sung Ha Park[2,3], Kyeong Kyu Kim*[1,3]

[¶] *These two authors contributed equally to this work.*

[1]*Department of Molecular Cell Biology, Sungkyunkwan University School of Medicine, Suwon 16419, Korea*

[2] *Departments of Physics, Sungkyunkwan University, Suwon 16419, Korea*

[3] *Sungkyunkwan Advanced Institute of Nanotechnology (SAINT), Sungkyunkwan University, Suwon 16419, Korea*

Supporting information

Content:

Supplementary Results and Figures

1. X-Ray photoelectron spectroscopy (XPS) study of bare and aminated Fe_3O_4@ SiO_2-NH_2 MCSNPs
2. pH dependent zeta potential study of MNPs
3. Assessment of expression and bioluminescence of *luxCDABE* in *UPEC* and *MRSA*
4. Genotype characterization of *fimA* deletion-insertion mutant and *fimA* complemented *UPEC* strain
5. Binding of MCSNPs to *MRSA* cell surface
6. Inhibition of *MRSA* biofilm formation using varying concentrations of MCSNPs
7. Live/dead staining of MDR *UPEC* during RMT
8. *UPEC* surface topography without or with MCSNPs at 25℃

Supplementary Tables

Supplementary References

1. **X-Ray photoelectron spectroscopy (XPS) study of bare and aminated Fe_3O_4@ SiO_2-NH_2 MCSNPs**

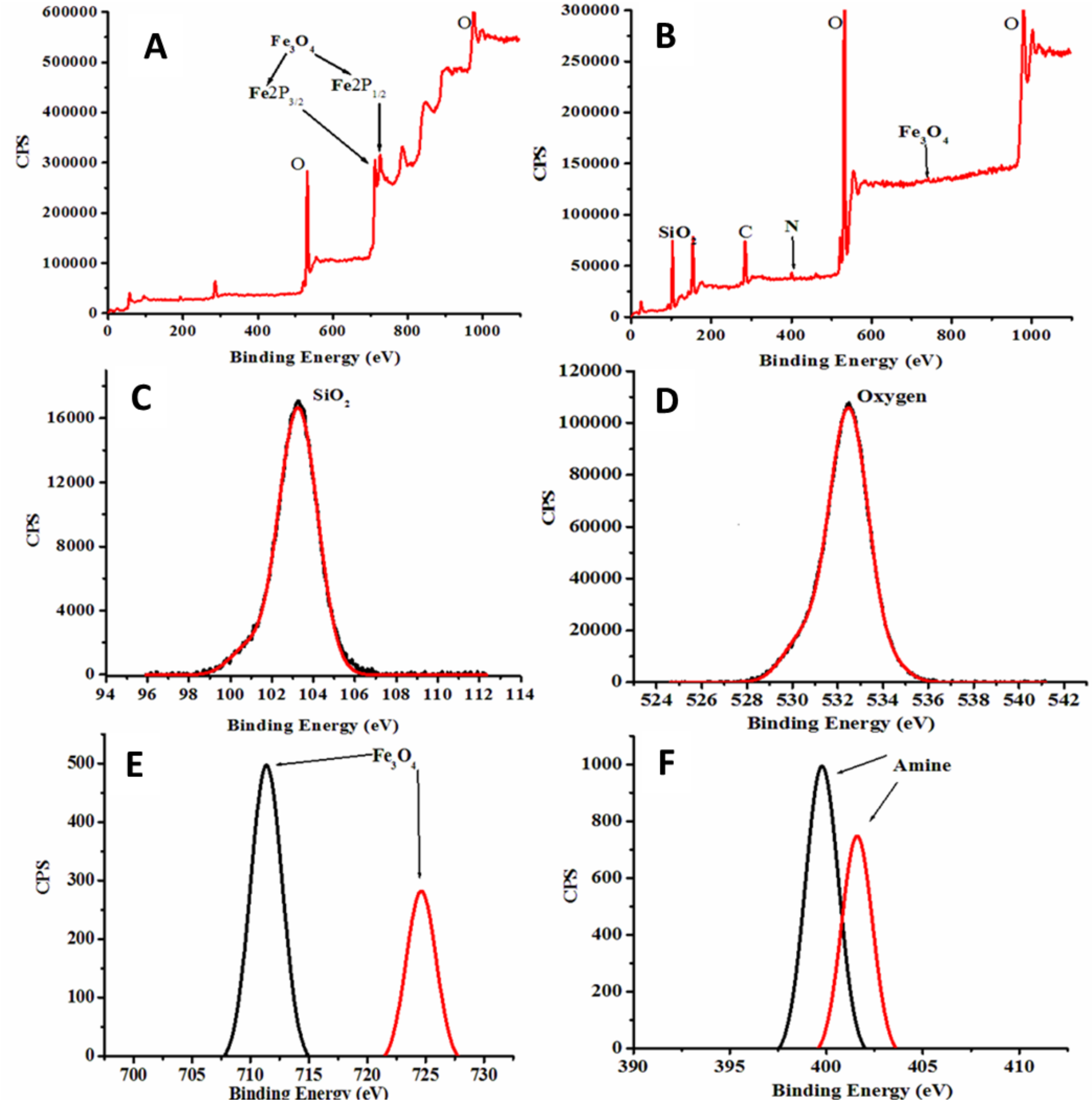

Figure S1. X-Ray photoelectron spectroscopy (XPS) study of bare and aminated Fe_3O_4@ SiO_2-NH_2 MCSNPs. Various spectra show **(A)** Fe electronic configuration peaks at 723 and 710 eV; **(B)** Si-O peaks observed at 103 eV; **(C)** amine-functionalization (NH_2) on the surface of Fe_3O_4–SiO_2 core-shell was observed at 399 eV; and **(C-F)** exact intensities of each peak corresponding to Si, O, Fe, and NH_3.

2. pH dependent zeta potential of MNPs

Zeta potential analysis was conducted to examine the surface charge reversal after the addition of silica and amine coatings on the Fe_3O_4. The isoelectric point (IEP) of bare Fe_3O_4 nanoparticles was found to be 6.5 (**Figure S2A**). After silica coating, the IEP value is shifted towards lower pH (pH 4.2) due to creation of more negative charge through dissociation of protons from the silanol group (Si-OH) of silica.[1] The zeta potentials of the negatively charged silica coated Fe_3O_4 core-shell MNP was changed to highly positive at pH ranging from 2 to 10 upon modification using positively charged APTES molecules (**Figure S2A**). The observed IEP for $Fe_3O_4@SiO_2-NH_2$ system was found to be 10.4 and APTES had IEP values of ~10.05 due to cationic amino groups.[2] The effect of phosphate buffer saline (PBS) at different pH ranges (pH 7.0-7.8) on zeta potential of $Fe_3O_4@SiO_2-NH_2$ was studied (**Figure S2 B**).

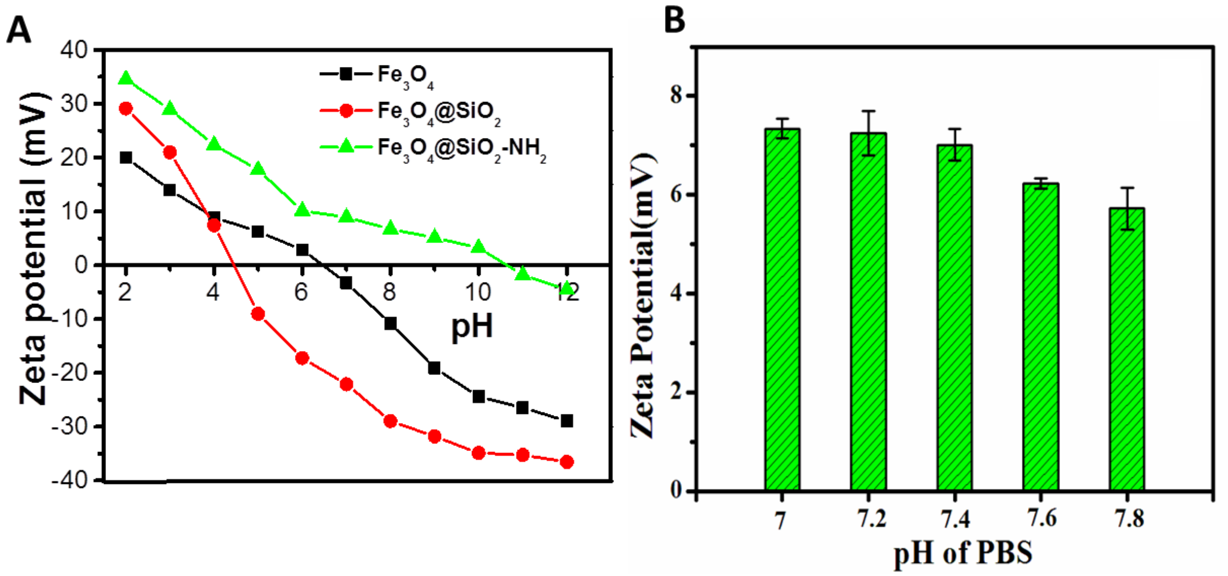

Figure S2. pH dependent zeta potential study of nanoparticles. (A) Zeta potential of Fe_3O_4, $Fe_3O_4@SiO_2$, and $Fe_3O_4@SiO_2-NH_2$ in water; **(B)** effect of pH in PBS on zeta potential of $Fe_3O_4@SiO_2-NH_2$. $Fe_3O_4@SiO_2-NH_2$ showed positive zeta potential (positive charge) at pH 7.4.

3. Assessment of expression and bioluminescence of *luxCDABE* in *UPEC* and *MRSA*

The genetically engineered bioluminescent strains *of E. coli CFT073* (*UPEC'*) and *S. aureus* USA300 FPR3757 (*MRSA'*) were created as described in materials and methods section of the main text of the article. The bioluminescent phenotype was tested both in liquid cultures and bacterial streaks on plates as shown in Figure S3.

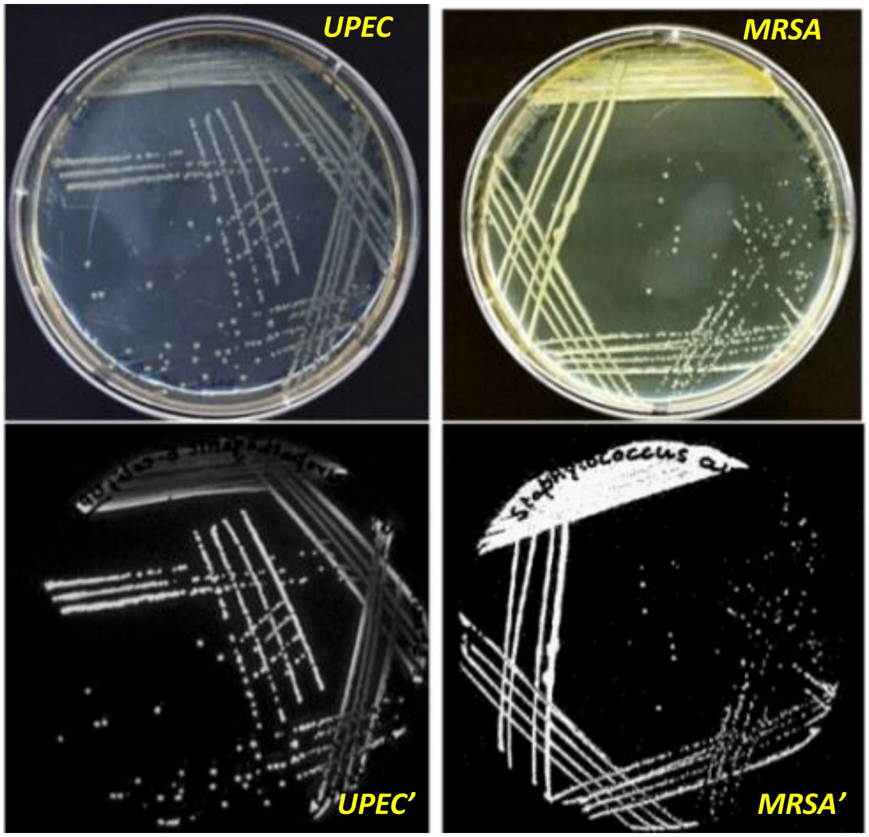

Figure S3. *UPEC* and *MRSA* expressing the bioluminescence gene *luxCDABE*. Bright field photograph of genetically engineered *UPEC* and *MRSA* showing bioluminescent colonies by dark field imaging (*UPEC'* and *MRSA'* on plates).

4. **Characterizing the genotype of *fimA* deletion insertion mutant and *fimA* complemented strain of *E. coli* CFT073 (*UPEC*)**

PCR amplification using kanamycin resistance marker primers pair (*km*^{r}*_fwd* and *km*^{r}*_rev*, **Table S1**) on genomic DNA of wild type *UPEC* showed no amplification of kanamycin cassette, while Δ*fimAΩkm*^{r} knockout and Δ*fimAΩkm*^{r} knockout complemented strains showed amplification of about 1 kb kanamycin cassette. The PCR amplification of *fimA* gene using *fimA* open reading frame primers pairs (*fimA_comp_fwd* and *rev;* **Table S1**) and *fimA* internal primer pairs (*fimA_int_fwd*, and *rev;* **Table S1**) on genomic DNA of wild type *UPEC* and Δ*fimAΩkm*^{r} knockout complemented strains showed the amplification of *fimA* gene. Amplification of *fimA* gene was not observed in the Δ*fimAΩkm*^{r} knockout strain. The absence of *fimA* and presence of kanamycin resistance gene in Δ*fimAΩkm*^{r} confirmed the deletion of *fimA* gene and insertion of *km* gene. The *fimA* deletion and *km* cassette insertion at the same locus was confirmed by amplification of PCR product using *FimAFRT KO_fwd and FimAFRT KO_rev* primer pairs (**Table S1**) and DNA sequencing.

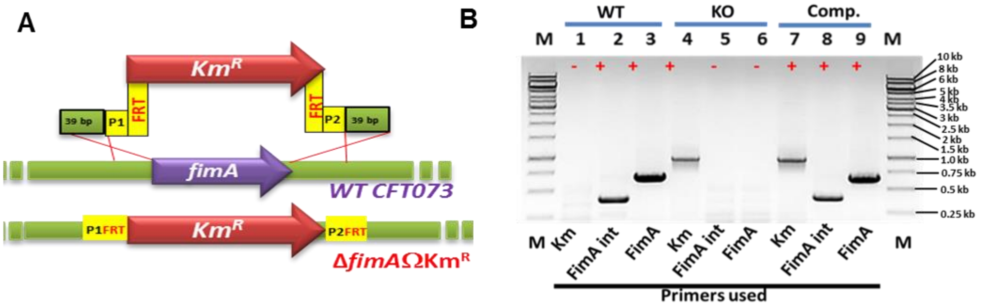

Figure S4. Red recombinase system mediated deletion-insertion of *fimA*, and genotypic characterization of Δ*fimA* deletion-insertion mutant and Δ*fimA* complemented strain of *UPEC*. (A) Schematic diagram showing the knock-out of *fimA* gene encoding fimbriae using red recombinase system; (**B**) Electrophoretogram of genotype verification of wild type *UPEC*, Δ*fimA* deletion-insertion mutant (Δ*fimAΩkm*^{r}) and complementation strains (Δ*fimAΩkm*^{r}*::pQE30fimA*) using kanamycin resistance gene primers (*km*), *fimA* internal primers (*fimA_int*) and *fimA* open reading frame (ORF) primer (*fimA*). No amplification of kanamycin was observed in wild type (lane 1) while the amplification of kanamycin cassette was observed in deletion-insertion mutant (Δ*fimAΩKm*^{r}) (lane 4) and its complemented strain (lane 7). No amplification of the *fimA* orf was seen in Δ*fimAΩkm*^{r} (lane 5 and 6) while the wild type (lane 2 and 3) and complemented strains (lane 8 and 9) showed the amplification of *fimA* gene.

5. Binding of MCSNPs to the cell surface of *MRSA*

MRSA cells were treated with 1 mg/mL MCSNP and subjected to scanning electron microscopy as described in the materials and methods section of the main text of the article. The SEM image shows the binding of numerous MCSNPs to the surface of *MRSA*. The magnetic capture of *MRSA* was found to be concentration dependent and about 96% of MRSA can be captured at 6 mg/mL MCSNPs, which is equivalent to 6 pg/cell.

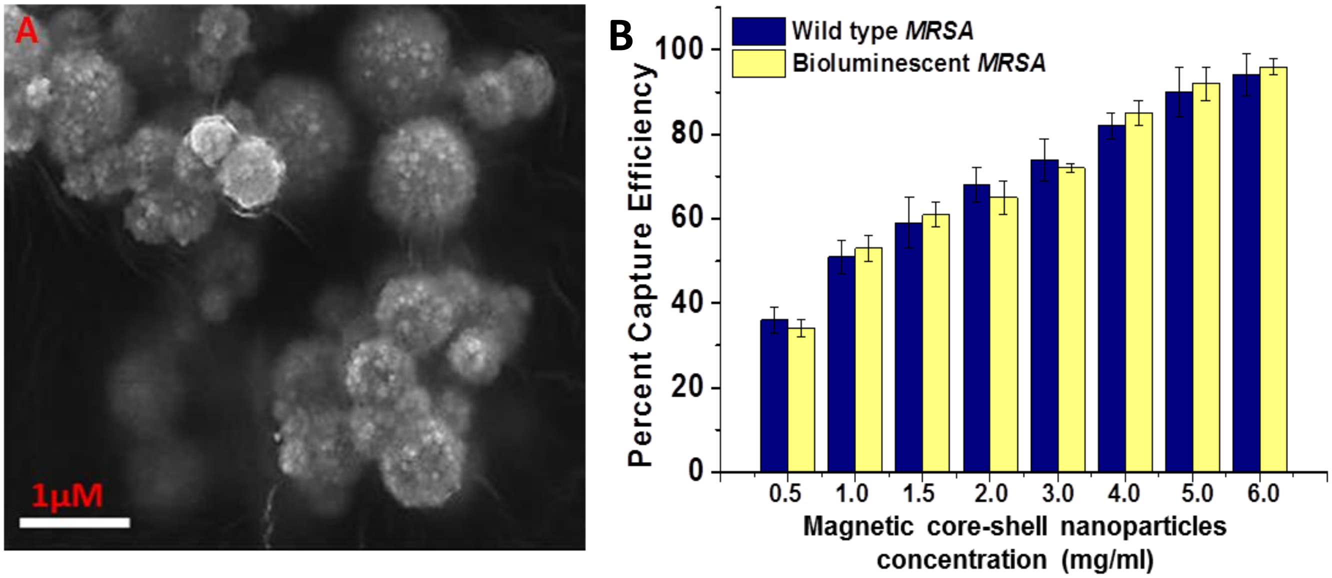

Figure S5. Interaction of MCSNP with *MRSA* cell surface and MCSNP mediated magnetic capture of *MRSA*. (A) SEM image of MCSNP-trapped *MRSA* at 1 mg/mL NP concentration, showing interaction of MCSNP to *MRSA* cell surface; (**B**) Magnetic capture efficiency of wild type *MRSA* and the genetically engineered bioluminescent strain assessed by measuring optical density and bioluminescence of remaining bacteria in suspension after magnetic capture of MCSNP-trapped bacteria.

6. **Inhibition of *Staphylococcus aureus* USA300 (*MRSA*) biofilm using varying concentrations of MCSNPs.**

MRSA biofilms were stained with fluorescein diacetate (FDA) only. The FDA stained biofilms were observed under confocal laser microscope to measure the biofilm thickness without (**Figure S6A I**) or with MCSNP treatment at varying concentrations (100, 200. and 500 µg mL^{-1}) (**Figure S6A II-IV and B-B'**). MCSNP treatment inhibited *MRSA* biofilm in a concentration-dependent manner, and biofilm was completely inhibited at 1 mg/mL MSCNPs **(Figure S6C)**.

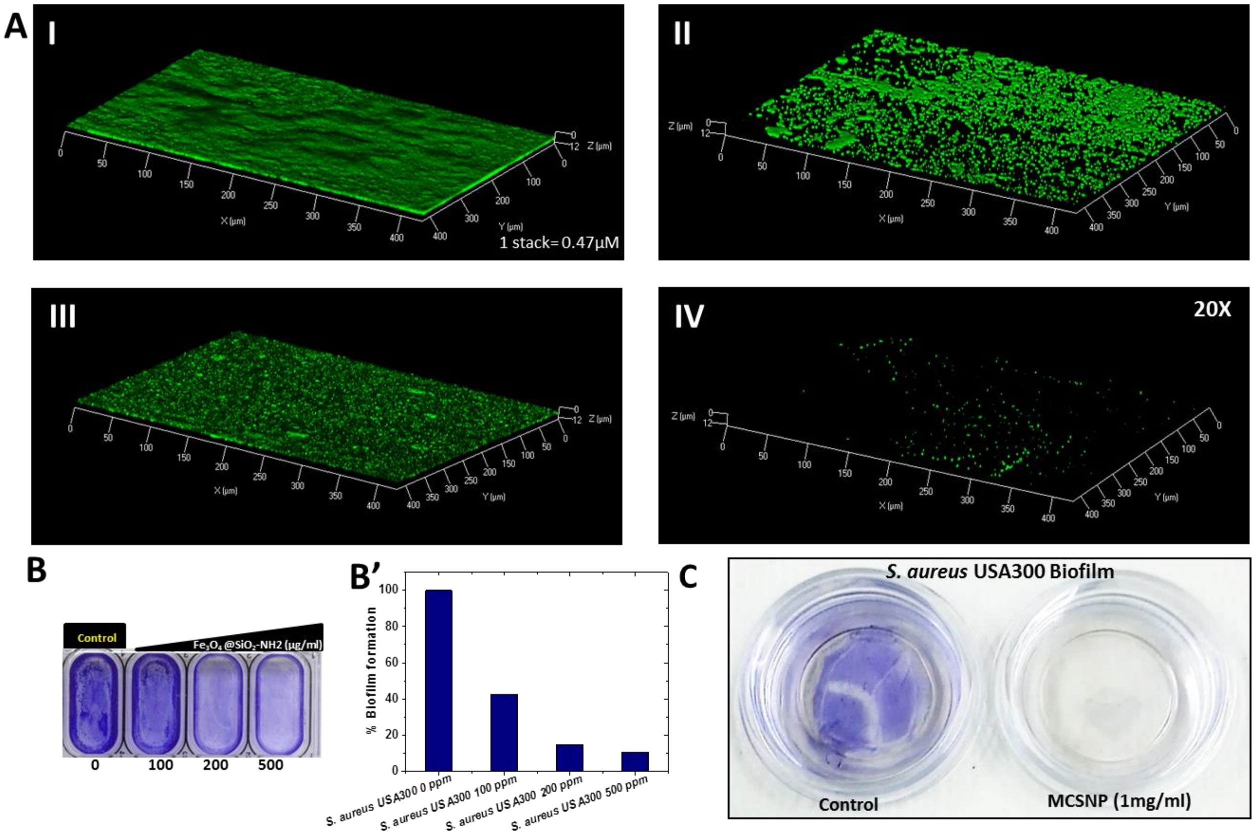

Figure S6. *MRSA* biofilm inhibition using varying concentrations of MCSNPs. (A) Confocal image of *MRSA* biofilm stained with fluorescein diacetate (FDA). **(a)** Control biofilm without MCSNPs, (**b-d**) Inhibition of MRSA biofilm at 100 (b), 200 (c), and 500 (d) µg/mL MCSNPs. **(B)** Images of crystal violet (CV) stained *MRSA* biofilm of control (0 µg/ml) and with varying concentrations of MCSNPs (100, 200, and 500 µg/mL) in chambered slide. **(B')** Quantitative estimation of CV stained biofilm by spectrophotometric analysis at 550 nm (A_{550}) verified the concentration dependent inhibition of *MRSA* biofilm by MCSNPs. **(C)** CV stained biofilm of MRSA control (no MSCNPs) and with MCSNPs at 1 mg/mL).

7. **Live/dead staining of MDR *UPEC* during RMT.**

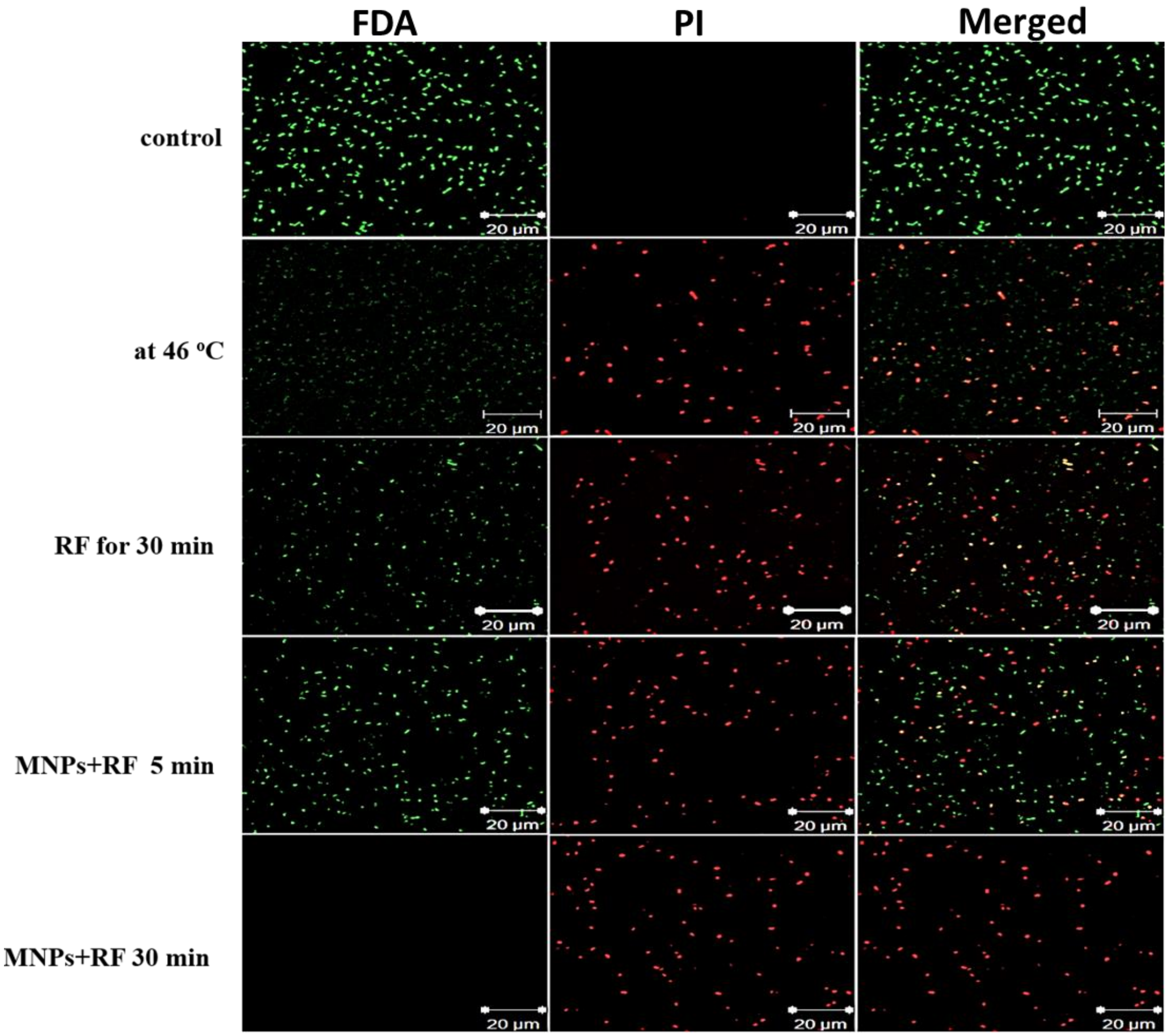

Figure S7. Live/dead staining of *E. coli* CFT073 during RMT. Live/dead confocal imaging under control and treated conditions using fluorescein diacetate (FDA) and propidium iodide (PI) staining where live and dead bacteria are visible in green and red, respectively. Various panels in the image are for control (bacteria at room temperature), 46℃ for 30 min, and radiofrequency (RF) treatment for 30 min and two time points, 5 min and 30 min, for the RF treatment coupled with MCSNPs (RMT). The RMT sample shows no traces of live (green bacteria) after 30 min of treatment.

8. Surface topography of UPEC with and without MCSNP treatment at 25°C

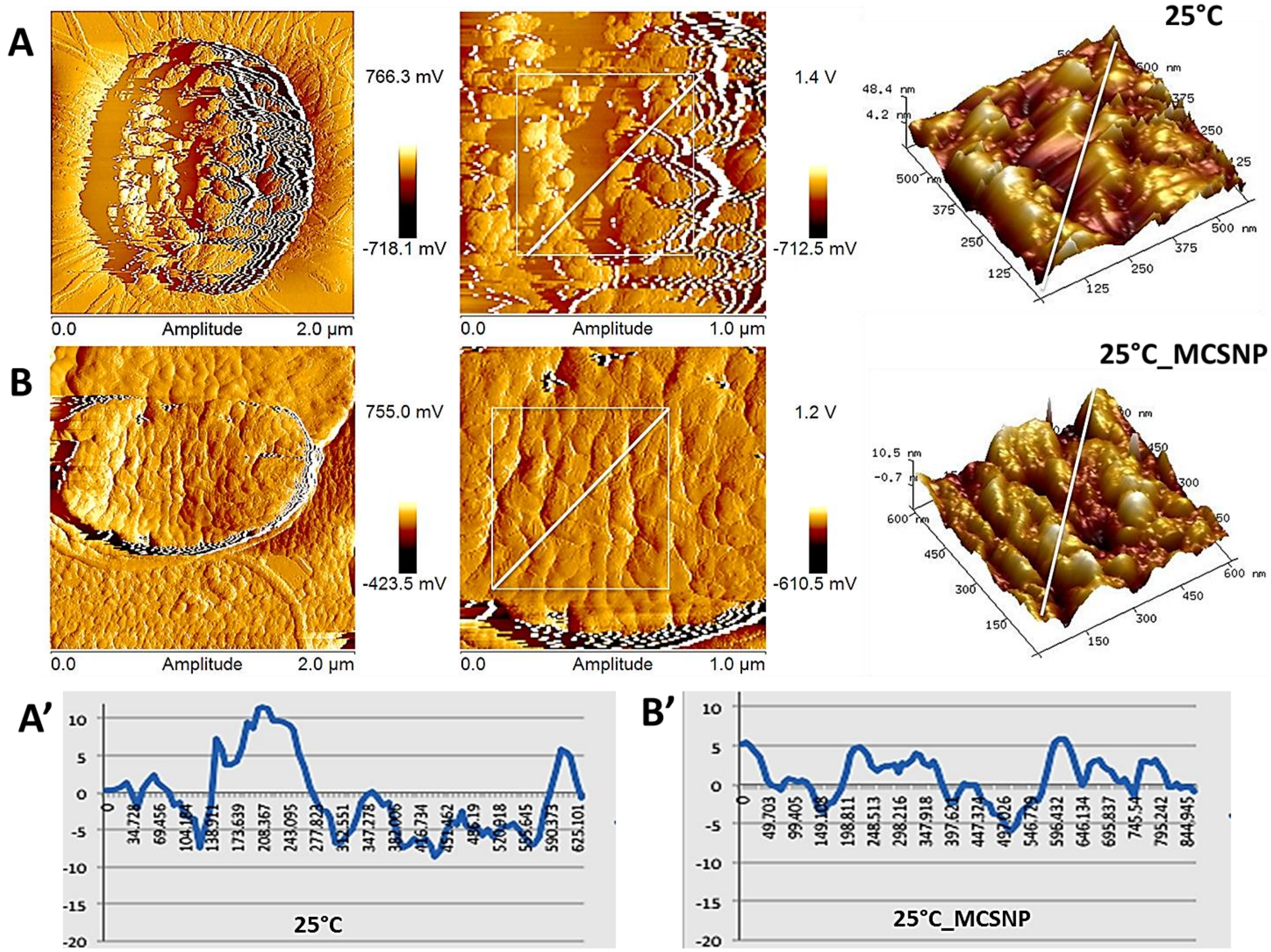

Figure S8. Topographic analysis of *E. coli* CFT073 surface at room temperature (25°C) to observe the effect of positively charged MCSNPs. (A-A') *E. coli* CFT073 without MCSNPs treatment and (**A**) and surface analysis (**A'**) (**B-B'**) MCSNPs treatment for 30 min (**B**) and surface analysis (**B'**). Both samples (A and B) were washed twice using deionized milliQ water at room temperature before AFM analysis. No significant differences were observed on membrane topography upon MCSNP treatment at room temperature (25°C).

Supplementary Tables

Table S1. Primers, plasmids used in this study

**Primers**	**Sequences**	**Source/reference**
1. *fimAFRT KO_fwd*	5'-TGTGCAGTGTTGGCAGTCAAACTCGTTGACAAAACAAAGTGGAAGTTCCTATA CTTTCTAGAGAATAGGAACTTCGGAATAG-3'	This study
2. *fimAFRT KO_rev*	5'-CATCCCTGCCCGTAATGACGTCCCTGAACCTAGGTAGGTTAGAAGTTCCTATTCT CTAGAAAGTATAGGAACTTCAGAGCGC-3'	This study
3. *fimA_comp_fwd*	5'-ACCATAGAATTCAGGAGAGGATCCGTGTACAGAACGACTGC-3'	This study
4. *fimA_comp_rev*	5'-ACCATAAAGCTTTTATTGATACTGAACCTTGAAGG-3'	This study
5. *fimA_int_fwd*	5'-CGTTCACTTTAAAGGGGAAG-3'	This study
6. *fimA_int_rev*	5'-GCGCCTGGAACGGAATGGTG-3'	This study
7. *kmR_fwd*	5'-AACGGTGCTGACCCCGGATGAATGTCAG-3'	This study
8. *kmR_rev*	5'-TCAGAAGAACTCGTCAAGAAGGCGATAG-3'	This study
**Plasmids**		
1. *pluxEGFP*	EGFP cloned under P_{luxI} promoter in pCDF-Duet-1 vector, sm^r	Lab collection
2. *pKD46*	Lambda red recombinase expressing vector under arabinose inducible promoter (P_{araB}), amp^r, repA101ts	[3]
3. *pKD4*	Kanamycin resistance gene flanked by FRT sites, amp^r, km^r,	[3]
4. *pQE30*	Expression under T5 promoter His-tag at the N terminus of the expressed protein amp^r, cm^r	Qiagen
5. *pQE30fimA*	*fimA* orf cloned at *EcoRI-HinDIII* sites	This study
6. *pGEN-luxCDABE*	*luxCDABE* constitutively expressing under em7 synthetic promoter	[4]
7. *pRP1195*	Integration site USA300HOU_1102 pseudogene, *luxBADCE* expression under P_{gapA}	[5]

Table S2. **Strains used in this study**

	Strains	Characteristics	Source/references
a.	*E. coli* DH5α	*F- endA1 glnV44 thi-1 recA1 relA1 gyrA96 deoRnupG Φ80dlacZΔM15 Δ(lacZYA-argF)U169, hsdR17(rK- mK+), λ–*	Lab collection
b.	*E. coli* CFT073 (*UPEC*)	Pyelonephritis isolate, *fim+ pap+ hly+*	ATCC
c.	Δ*fimAΩkm*^{r}	Deletion-insertion mutant of *fimA*, *km*^{r}	This Study
d.	Δ*fimAΩkm*^{r}::pQE30*fimA*	Complementation of *fimA* deletion mutant, *km*^{r}, *amp*^{r} and *cm*^{r}	This Study
e.	Ec_CFT073_EGFP	*E. coli* CFT073 expressing EGFP under P_{luxI}, *sm*^{r}	This Study
f.	Bioluminescent *UPEC'*	*E. coli* CFT073 expressing em7-*lux*, *amp*^{r}	This Study
g.	*SAP140*	*RN4220* with freely replicating pRP1195 (temperature sensitive)	[5]
h.	*S. aureus* USA300 FPR3757 (*MRSA*)	wild type *Staphylococcus aureus* USA300 FPR3757 strain	NARSA
i.	Bioluminescent *MRSA'*	*MRSA* stably expressing *luxBADCE*	This Study

Supplementary References

1 Reddy, P. M., Chang, K. C., Liu, Z. J., Chen, C. T. & Ho, Y. P. Functionalized magnetic iron oxide (Fe_3O_4) nanoparticles for capturing Gram-positive and Gram-negative bacteria. *J. Biomed. Nanotechnol.* **10**, 1429-1439 (2014).

2 Bini, R. A., Marques, R. F. C., Santos, F. J., Chaker, J. A. & Jafelicci, M. Synthesis and functionalization of magnetite nanoparticles with different amino-functional alkoxysilanes. *J. Magn. Magn. Mater.* **324**, 534-539 (2012).

3 Datsenko, K. A. & Wanner, B. L. One-step inactivation of chromosomal genes in Escherichia coli K-12 using PCR products. *Proc. Natl. Acad. Sci. U S A* **97**, 6640-6645 (2000).

4 Lane, M. C., Alteri, C. J., Smith, S. N. & Mobley, H. L. Expression of flagella is coincident with uropathogenic Escherichia coli ascension to the upper urinary tract. *Proc. Natl. Acad. Sci. U S A* **104**, 16669-16674 (2007).

5 Plaut, R. D., Mocca, C. P., Prabhakara, R., Merkel, T. J. & Stibitz, S. Stably luminescent *Staphylococcus aureus* clinical strains for use in bioluminescent imaging. *PLoS One* **8**, e59232 (2013).
